# Health related quality of life in Malaysian children with thalassaemia

**DOI:** 10.1186/1477-7525-4-39

**Published:** 2006-07-02

**Authors:** Adriana Ismail, Michael J Campbell, Hishamshah Mohd Ibrahim, Georgina L Jones

**Affiliations:** 1 Institute of General Practice and Primary Care, Community Sciences Centre, University of Sheffield, Northern General Hospital, Sheffield S5 7AU, UK; 2 Institute of Paediatrics, Hospital Kuala Lumpur, Kuala Lumpur 50580, Malaysia

## Abstract

**Background:**

Health Related Quality of Life (HRQoL) studies on children with chronic illness such as thalassaemia are limited. We conducted the first study to investigate if children with thalassaemia have a lower quality of life in the four dimensions as measured using the PedsQL 4.0 generic Scale Score: physical, emotional, social and role (school) functioning compared to the healthy controls allowing for age, gender, ethnicity and household income.

**Methods:**

The PedsQL 4.0 was administered to children receiving blood transfusions and treatments at Hospital Kuala Lumpur, Malaysia using PedsQL 4.0 generic Scale Score. Accordingly, the questionnaire was also administered to a control group of healthy school children. Socio-demographic data were also collected from patients and controls using an interview schedule designed for the study.

**Results:**

Of the 96 thalassaemia patients approached, 78 gave consent to be interviewed giving a response rate of 81.3%. Out of 235 healthy controls approached, all agreed to participate giving a response rate of 100%. The mean age for the patients and schoolchildren is 11.9 and 13.2 years respectively. The age range for the patients and the schoolchildren is between 5 to 18 years and 7 to 18 years respectively. After controlling for age and demographic background, the thalassaemia patients reported having significantly lower quality of life than the healthy controls.

**Conclusion:**

Thalassaemia has a negative impact on perceived physical, emotional, social and school functioning in thalassaemia patients which was also found to be worse than the children's healthy counterparts. Continuing support of free desferal from the Ministry of Health should be given to these patients. More understanding and support especially from health authorities, school authorities and the society is essential to enhance their quality of life.

## Background

Thalassaemia is a genetic blood disorder which can be fatal if proper treatment is not received. It is characterised by partial or no production of alpha or beta globin chains which form part of the structure of the haemoglobin in the red blood cells [1]. Thalassaemia is an increasingly serious public health problem throughout the Mediterranean region, the Middle East, the Indian subcontinent and South East Asia [2,3]. In Malaysia, it occurs mainly in the Malays and Chinese Malaysians [4]. The Ministry of Health of Malaysia estimated that between 150 and 350 babies are born with thalassaemia each year [5]. George et al [4] estimated that there are about 5,600 patients with blood transfusion dependent beta thalassaemia in Malaysia.

Children with thalassaemia appear well at birth but develop anaemia that becomes progressively worse due to the partial or total absence of haemoglobin. If left untreated, this can result in early deaths [2,6]. For those children that do survive, this condition has serious implications for the health related quality of life of children. The children typically, have to undergo blood transfusions at least once a month depending on the severity of the illness. This means they have to attend the hospital which sometimes takes a whole day. They also have to get desferal injections for iron chelation therapy to remove excess iron in their blood from the blood transfusions [2,6,7].

Health Related Quality of Life (HRQoL) measurement is a multidimensional concept that focuses on the impact of the disease and its treatment on the well being of an individual. The measures are seen as ways of capturing patients' perspectives of their disease and treatment, their perceived need for healthcare and their preferences for treatment and disease outcomes [8]. A systematic review, however, reports of limited use of HRQoL measures in paediatric clinical trials or clinic practice [9]. The review identified 18 trials which include assessment of HRQoL on a variety of paediatric diseases but none on Thalassaemia. This is surprising given the nature of the illness.

An extensive and rigorous search on websites and electronic databases by the team found one paper on the HRQoL of adults with thalassaemia and one for children. Most of the research on thalassaemia involves interviews with patients, carers, nurses and doctors and the focus was on coping strategies for thalassaemia patients and their parents, the attitudes and expectations of these patients, counselling strategies for thalassaemia patients, parents and siblings and screening programmes for thalassaemia. A recent study on adults with thalassaemia suggests that treatment and cultural differences did not have a major effect on the Quality of life in Cypriot thalassaemia patients [10]. The research was conducted in a well established resource centre and 212 adult patients were involved. Another study on children compared the quality of life of patients with thalassaemia intermedia to thalassaemia major and discovered that transfusion – independent thalassaemia patients also suffer impairments in their quality of life [11]. The team suggested that all patients with thalassaemia undergo QoL assessment so that interventions focused on affected domain can be implemented. As a result of the limited research on the HRQoL of thalassaemia patients in particular children, the aim of this study was to investigate if the HRQoL thalassaemia patients is worse than the HRQoL of healthy children in the essential core domains for pediatric HRQoL measurement: physical functioning, emotional functioning, social functioning and school functioning.

## Methods

This was a cross sectional design study in Kuala Lumpur, Malaysia. For sample size, the number of cases was limited by availability to less than 100 and we oversampled the controls to maintain power. Norman et al [12] have shown that for many quality of life scores an effect size of about 0.5 SDs is reasonable and for this, one should need 64 cases and 64 controls for at least 80% power at 5% significance level [13].

The patients interviewed were receiving blood transfusions and treatments at the Paediatric Institute, Hospital Kuala Lumpur in the period May 2005 to August 2005. The patients were approached as they come in for their blood transfusions scheduled at the Institute. The study was approved by the Hospital Ethics Committee. Children with thalassaemia come in to the Day Care Centre at the Institute for their blood transfusions and treatments. Most of them have been getting their treatments at the Institute since they were diagnosed as having thalassaemia. Previously, patients had to buy their own desferal for their iron chelation therapy. Patients are known to miss their desferal injections because they could not afford to buy desferal. However, since December 2004, six months before this study was conducted, the Paediatric Institute was the first Government hospital in Malaysia to provide desferal to all patients getting treatment there. Further inclusion criteria were written informed consent, aged between 5 to 18 years and patients who could read, write and understand Malay, English or Mandarin.

For the healthy controls, the questionnaires were administered to children in the neighbouring schools between 7 to 18 years of age. Permission to administer the questionnaires was granted by the Economic Planning Unit at the Prime Minister's Department and the Ministry of Education of Malaysia. Thus this study investigates the HRQoL of children with thalassaemia six months after getting proper treatment (blood transfusions and desferal) at the Institute.

### Research instrument

HRQoL was assessed with the PedsQL 4.0 Generic Core Scales. This instrument has 23-items that are designed to measure the core dimensions of health as delineated by WHO. The PedsQoL 4.0 encompasses the essential core domains for paediatric HRQoL measurement: 1) Physical Functioning (8 items), 2) Emotional Functioning (5 items), 3) Social Functioning (5 items) and 4) School Functioning (5 items). It consists of developmentally appropriate forms for ages 2–4, 5–7, 8–12 and 13–18 years. The reliability, validity, responsiveness, and practicality of the PedsQL Generic Core Scales have been assessed in both physically healthy paediatric populations and in paediatric acute and chronic health conditions. The internal consistency reliability of the PedsQL 4.0 Generic Core Scale approached 0.90 for self-report [14-16]. Missing data were also minimal. Item response distributions were across the full scale range, with no floor effects, and minimal ceiling effects. The validity of the PedsQL Generic Core Scales has been demonstrated through known group comparisons, and correlations with other measures of disease burden. User agreement was signed with MAPI Research Institute, Lyon, France prior to using the questionnaires. PedsQL 4.0 Generic Core Scales has also been translated into many languages and we use the UK English, Malaysian Language and Chinese Mandarin versions as appropriate.

### Statistical analysis

The assumptions of normality and homoscedasticity were investigated and found plausible [17,18]. Differences in the variables between thalassaemia patients and healthy controls were tested using the independent t-test and Chi Square Test. Since the controls are slightly older, we also conducted a matched control analysis on the two groups by age and gender. A paired sample t-test was used to test the differences between the pairs. We further exclude patients who have had bone marrow transplants in our analysis to compare results. We then used multiple linear regressions to compare the patients and controls for the different domains in PedsQL 4.0, adjusting for age, gender, ethnicity and household income. We also tested for interactions between these covariates on the outcome variable. For all the tests conducted, a p value of ≤ 0.05 is considered statistically significant.

## Results

A total of 96 patients receiving treatments at the Institute were approached out of 140 on the record. 14 (14.6%) did not fit inclusion criteria (age, did not understand Malay, Mandarin or English), 1 refused (1.0%), 3 (3.1%) quit halfway and 78 completed the interview. This gives a response rate of 81.3% for the thalassaemia patients. The three patients who quit halfway were restless and decided not to continue with the interviews. Of the 235 questionnaires distributed to the healthy controls, all of them answered and responded to the questionnaires, giving a 100% response rate.

Table 1 summarises the demographic variables of the 78 thalassaemia patients and 235 healthy controls. The healthy controls are slightly older, have higher education and are better off than the patient group. The mean age of the healthy controls was 13.2 years old and the age range was between 7 to 18 years old. The mean age of the patients is 11.9 years old and the age range was between 5 to 18 years old. 37 out of 78 patients (47.4%) came from families with household income of less than RM1500. Of the 235 participants in healthy group, only 68 (29%) came from families with household income of less than RM1500. Both the thalassaemia patients and controls came from a limited number of ethnic backgrounds: Malay, Chinese, Indian and others which include Orang Asli and Indonesian Heritage. Nevertheless, the majority for both thalassaemia children and healthy controls were Malays. In terms of gender, the thalassaemia group had more males than the control group but this was not statistically significant (p = 0.311).

**Table 1:** Demographic Characteristics of Thalassaemia patients

	**Thalassaemia Patients (n = 78)**	**Healthy Controls (n = 235)**	**P values**
**Age, y+**	11.95 (4.3)	13.2 (2.8)	p = 0.004
			
Gender*			p = 0.311
Female	34 (43.6)	118 (50.2)	
**Range**			
5–7 years	8 (18.2)	7 (6.0)	
8–12 years	19 (43.2)	47 (40.2)	
13–18 years	17 (38.6)	63 (53.8)	
Male	44 (56.4)	117 (49.8)	
**Range**			
5–7 years	6 (17.6)	6 (5.1)	
8–12 years	14 (41.2)	21 (17.8)	
13–18 years	14 (41.2)	91 (77.1)	
			
Ethnicity*			
Malay	56 (71.8)	136 (57.9)	p = 0.001
Chinese	16 (20.5)	53 (22.5)	
Indian	1 (1.3)	40 (17.0)	
Others	5 (6.4)	6 (2.6)	
			
Religion*			p = 0.001
Islam	57 (73.1)	138 (58.7)	
Buddha	14 (17.9)	44 (18.7)	
Hindu	1 (1.3)	34 (14.5)	
Christian	2 (2.6)	17 (7.2)	
Others	4 (5.1)	2 (0.9)	
			
Educational* Level			p < 0.001
Not at School	4 (5.1)	0 (0.0)	
Pre School	5 (6.4)	0 (0.0)	
Primary (1–6)	38 (48.7)	81 (34.5)	
Secondary (1–5)	18 (23.1)	150 (63.8)	
Sixth Form	2 (2.6)	4 (1.7)	
Finish School	11 (14.1)	0 (0.0)	
			
Household* Income (RM)			p < 0.001
<500	2 (2.6)	3 (1.3)	
500–1000	16 (20.4)	27 (11.5)	
1000–1500	19 (24.4)	38 (16.2)	
1500–2000	19 (24.4)	25 (10.6)	
>2000	21 (26.9)	138 (58.7)	
Missing	1 (1.3)	4 (1.7)	

* Values are presented as number (percentage) unless otherwise indicated. + Data given as Mean (SD). RM is an acronym for Malaysian Ringgit.

The different types of thalassaemia and medical procedures are given in Table 2. Patients were more likely to have beta thalassaemia major, than HbE/beta thalassaemia and beta thalassaemia intermedia. Most of the patients (n = 74) have not had bone marrow transplants and have not undergone splenectomy (n = 56). 84.8%, 88.9% and 56.5% of the beta thalassaemia major patients, beta thalassaemia intermedia patients and HbE beta thalassaemia patients are respectively transfusion dependent. We also calculated the mean serum ferritin level and the mean dosage for the three groups. The mean serum ferritin level is 4739.45 mcg/L and the mean desferal dosage is 40 mg/kg/day given for 5 days a week.

**Table 2:** Thalassaemia types and medical procedures among Thalassaemia patients

**Types of Thalassaemia**	**All (n = 78)**	**Number with >8 blood transfusions per year (Transfusion Dependent)**
1. Beta Thalassaemia Major	46 (59.0)	39 (84.8)
2. Beta Thalassaemia Intermedia	9 (11.5)	8 (88.9)
3. HbE Beta Thalassaemia	23 (29.5)	13 (56.5)
Other medical procedures		
4. Have had bone marrow transplant	4 (5.1)	
5. Have had splenectomy	22 (28.2)	

Values are presented as number (percentage).

Table 3 lists mean scores of the four PedsQL 4.0 subscales and their summarised Psychosocial Health Summary (PCHS) and Total Summary Score (TSS) for the thalassaemia patients and the differences of these scores compared to the healthy controls. The PCHS is the average of the Emotional, Social and School Functioning, and the TSS is the average scores of all items. The scores of three of the PedsQL domain (Physical, Social and School functioning) are statistically significantly lower than those of the healthy control with p < 0.05. The Thalassaemia subscales score for the three domains are about 10% to 24% lower than the healthy controls. The Thalassaemia subscale for Emotional Functioning is lower by 2.3% and it is found to be not statistically significant with p > 0.05. The scores for emotional functioning of thalassaemia patients with mean of 68.14 and sd of 17.22 is low in comparison to scores for emotional functioning of chronically ill children in other studies; for example, Varni et al [15] reported that the mean (sd) for emotional functioning of oncology patients which include acute lymphocytic leukaemia, brain tumour, non Hodgkin lymphoma, Hodgkin lymphoma and other cancer was 71.83 (21.44). Upton et al [19] later reported the means (sd) for emotional functioning of asthma, cancer and diabetic patients were 70.66 (20.06), 73.56 (18.39) and 78.85 (18.28) correspondingly. Unexpectedly, the score for emotional functioning of healthy controls in this study is equivalently low; mean (sd) is 69.72(17.71) and it is lower than scores for healthy children in other studies, namely, Varni et al [15,16] reported means of 80.8 and 79.45 with sd of 19.64 and 18.00 respectively for healthy children in the US. Upton et al [19] reported mean of 78.49 with sd of 17.94 for healthy children in the UK. The PCHS and TSS scores for the thalassaemia are also about 12% to 14% lower than the healthy controls and these are found to be statistically significant (p < 0.05).

**Table 3:** Mean Unadjusted PedsQL 4.0 Generic Quality of Life Scores in Thalassaemia patients compared to Healthy Schoolchildren

**Domain**	**Thalassaemia Patients (n = 78)**	**Healthy Controls (n = 235)**	**Difference**	**95% Confidence Interval of the Difference**	**P-Value**
Physical Functioning	69.15 (16.45)	84.84 (13.04)	-15.69	(-19.75 to -11.63)	<0.001
Emotional Functioning	68.14 (17.22)	69.72 (17.71)	-1.58	(-6.10 to 2.94)	0.492
Social Functioning	74.29 (18.77)	82.36 (15.87)	-8.07	(-12.34 to -3.78)	<0.001
School Functioning	60.14 (16.41)	79.21 (15.75)	-19.08	(-23.27 to -14.88)	<0.001
Psychosocial Health Summary	67.58 (12.77)	77.09 (13.12)	-9.52	(-12.99 to -6.04)	<0.001
Total Summary Score	68.91 (12.12)	79.79 (11.60)	-10.88	(-14.11 to 7.65)	<0.001

Values are presented as Mean (SD)

The results of the matched control analysis are given in Table 4. Similar results to Table 3 were obtained. Physical functioning, social functioning and school functioning of thalassaemia patients are lower than the healthy controls. The emotional functioning of the patients is similar to previous results which is slightly lower than the healthy controls and again found to be not statistically significant.

**Table 4:** Results from matched study

**Domain**	**Thalassaemia Patients (n = 70)**	**Healthy Controls (n = 70)**	**Difference**	**95% Confidence Interval of the Difference**	**P-Value**
Physical Functioning	69.17 (15.34)	85.49 (12.67)	-16.43	(-21.19 to -11.67)	<0.001
Emotional Functioning	68.14 (16.75)	70.36 (16.86)	-2.21	(-6.89 to 2.47)	0.349
Social Functioning	73.29 (18.69)	82.21 (15.29)	-8.93	(-14.34 to -3.51)	<0.05
School Functioning	59.86 (16.60)	80.00 (14.40)	-20.14	(-25.53 to -14.76)	<0.05
Psychosocial Health Summary	67.39 (13.24)	77.58 (12.63)	-10.19	(-14.18 to -6.21)	<0.001
Total Summary Score	67.70 (12.49)	79.51 (11.90)	-11.81	(-15.63 to -8.00)	<0.001

Values are presented as Mean (SD)

We reran the analysis, excluding patients who have had bone marrow transplants in another match control analysis by age and gender and we obtained similar results to table 3.

**Figure 1:**
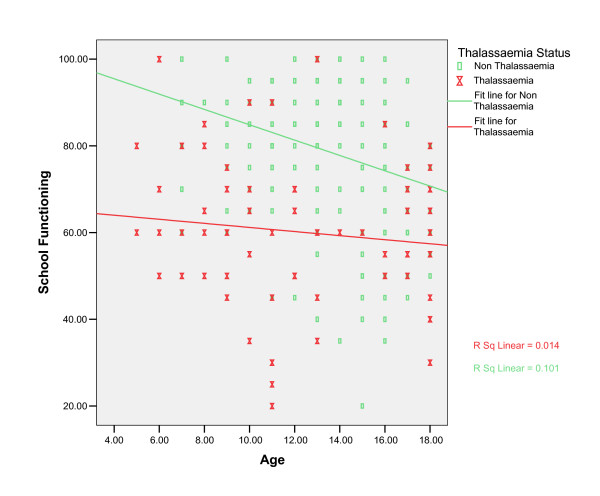
Impact of different Ages on School Functioning for Thalassaemia Children And Healthy Controls.

A multiple regression analysis was carried out to adjust for imbalances in age, sex, ethnicity and household income. The model is statistically significant for all domains except for Emotional Functioning (Table 5). An investigation of whether there were any interactions between predictors for the four scores, in which a significant difference showed only for school functioning did Age and Thalassaemia status interact. The fitted lines for School Functioning by age are shown in Figure 1 for Thalassaemia and Non Thalassaemia (Healthy Controls). It shows that the healthy controls start higher but decreases as they get older but the thalassaemia patients remain low throughout, so the effect of thalassaemia appears less for the older children. The difference in slope between the cases and controls is 1.298, 95%CI (0.193 to 2.402) with p value <0.001 and R adj = 0.263.

**Table 5:** Adjusted Difference in the Quality of Life Scores between Thalassaemia Children and Healthy Controls

**Domains**	**Regression Coefficients (Thalassaemia Status (ThalS)**	**95% CI**	**P values**	**Adjusted R squared**
Physical Functioning	-15.690	(-19.279 to -12.100)	<0.001	0.192
Emotional Functioning	-1.582	(-6.106 to 2.941)	0.4920	
Social Functioning	-8.067	(-12.345 to -3.789)	<0.001	0.039

## Discussion

The assessment of quality of life in children especially in children with chronic illness such as thalassaemia is particularly important. If they survive the illness, children not only have longer lives to lead compared to adults, but they are less able to voice their concerns and are more vulnerable than adults.

The use of self report health-related quality of life questionnaires in the assessment of thalassaemia patients can help identify the impact of the disease and its associated treatments from the children's perspective [20]. There have also been discussions among paediatricians and child health researchers about the importance of children's perspectives on their own health. A recent study found that children as young as six years old can adequately understand and accurately report their own health and well being [21]. Vincent and Higginson [22] stressed that any measurement of the quality of life of children should include questions on physical, social and psychological functioning of a child. They also emphasised that educational achievement, questions on sexuality, social and peer acceptance become significantly important as the children grow older especially during adolescent.

The PedsQL 4.0 Generic Core Scale self report was chosen by the team as the research instrument as it incorporates the dimensions necessary for measuring the HRQoL of paediatric population and has been tested for validity and reliability in both physically healthy paediatric populations and in paediatric acute and chronic health conditions [14-16].

There has not been previous study to compare the the HRQoL of thalassaemia patients to healthy children. This is the first study to provide evidence that the HRQoL of thalassaemia patients is lower than the HRQoL of healthy controls. The effect of thalassaemia on the HRQoL of these children is to reduce their physical, social and school functioning by about 10% to 24%. This was confirmed through our matched control analysis (Table 4). Although the unadjusted emotional functioning scores for thalassaemia patients appear lower than their healthy counterpart, a multiple regression analysis adjusting for age, gender, ethnicity and household income shows that the adjusted effect of thalassaemia on their emotional functioning is small and not statistically significant (Table 5). Pakbaz et al [11] earlier suggested that emotional functioning is one of the impaired domains of thalassaemia patients and this study shows that thalassaemia patients scored low in their emotional functioning (Tables 3 and 4). Interestingly, a study by Tsiantis et al [23] also suggests that thalassaemia patients have their own coping strategies in dealing with their life but these coping strategies are not examined in this study. It is also discovered that the healthy controls in this study score equally low in their emotional functioning. The score is lower than healthy samples in the United States [15,16] and in the UK [19]. The healthy controls are from urban areas in a developing country and are known to have hectic lifestyles. They go to school with heavy bags full of stationary and books that weigh sometimes up to 5 kg; involved in extra curricular activities (effectively stay at school up to 10 hours a day); early evening go for private tuitions. All these factors could aggravate their emotional functioning. This result hence warrants further research on healthy children across the country so a conclusive perspective could be arrived. As for this study, the result suggests that the difference between the thalassaemia children and the healthy controls is quite marginal and this is their own self-reported views within the context of Malaysia.

The results of the investigation of interaction terms in the multiple regression analysis show that school functioning of thalassaemia is affected. The school functioning of these children is lower than the controls and remain low throughout their life. This seems to prove that having to go to hospital for blood transfusions is one of the main reasons that thalassaemia patients are missing school and this is affecting their HRQoL [6,7]. Further studies should be conducted to investigate if missing school affects their performance in pursuing a good education as suggested earlier by Canatan et al [24]. The Psychosocial Health Summary scores of the thalassaemias are also lower than the healthy controls. This finding seems to support previous studies on psychosocial aspects of thalassemia that more psychosocial support should be given to thalassaemia patients [25,26].

The study also reveal that thalassaemia has different impact at different ages on school functioning. However, age does not have much impact on the other domains. Gender, ethnicity and household income seem not to have effect on the HRQoL of thalassaemia patients.

The limitation of this study is that it is conducted at only one Thalassaemia Day Care Centre in one hospital in Malaysia, and so, it might not be representative of the population of Thalassaemia patients in the country. Nevertheless, it is worth mentioning that the hospital is the national referral centre for thalassaemia and amongst the three big hospitals in Klang Valley, Malaysia with the greatest number of children with thalassaemia. It is also the first hospital in Malaysia to provide free desferal for the treatment of excess iron in the body of patients due to blood transfusion.

## Conclusion

From this study, we are able to conclude that the quality of life of thalassaemia patients is indeed much lower than the quality of life of healthy controls regardless of age, gender, ethnicity and household income. It is highly recommended that the Ministry of Health of Malaysia continue supporting free desferal to these patients. These patients also need the understanding and support especially from the health authorities, policy makers, Ministry of Education Malaysia, school authorities and the society to provide them better quality of life.

## Competing interests

The author(s) declare that they have no competing interests.

## Authors' contributions

**AI **was involved in the acquisition and collecting of data, the design, interpretation, analysis of data and drafting the manuscript,

**MJC **was involved in revising the paper for important intellectual content and in the design, interpretation and analysis of data.

**HMI **was involved in revising the content of data, in the acquisition of data and revising the paper for important intellectual content.

**GLJ **was involved in the design and revising the paper for important intellectual content
